# Toward Research-Informed Design Implications for Interventions Limiting Smartphone Use: Functionalities Review of Digital Well-being Apps

**DOI:** 10.2196/31730

**Published:** 2022-04-19

**Authors:** Sultan Almoallim, Corina Sas

**Affiliations:** 1 School of Computing and Communications Lancaster University Lancaster United Kingdom

**Keywords:** digital well-being, smartphone apps, tracking use, monitoring against set use limits, interventions for limiting use, barriers, design for friction, screen time, attention, self-regulation, mobile phone

## Abstract

**Background:**

Much research in human-computer interaction has focused on well-being and how it can be better supported through a range of technologies, from affective interfaces to mindfulness systems. At the same time, we have seen a growing number of commercial digital well-being apps. However, there has been limited scholarly work reviewing these apps.

**Objective:**

This paper aims to report on an autoethnographic study and functionality review of the 39 most popular commercial digital well-being apps on Google Play Store and 17 apps described in academic papers.

**Methods:**

From 1250 apps on Google Play Store, we selected 39 (3.12%) digital well-being apps, and from Google Scholar, we identified 17 papers describing academic apps. Both sets of digital well-being apps were analyzed through a review of their functionalities based on their descriptions. The commercial apps were also analyzed through autoethnography, wherein the first author interacted with them to understand how these functionalities work and how they may be experienced by users in their daily lives.

**Results:**

Our findings indicate that these apps focus mostly on limiting screen time, and we advanced a richer conversation about such apps, articulating the distinctions among monitoring use, tracking use against set limits, and 4 specific interventions supporting limited use.

**Conclusions:**

We conclude with 6 implications for designing digital well-being apps, namely calling to move beyond screen time and support the broader focus of digital well-being; supporting meaningful use rather than limiting meaningless use; leveraging (digital) navigation in design for friction; supporting collaborative interaction to limit phone overuse; supporting explicit, time-based visualizations for monitoring functionality; and supporting the ethical design of digital well-being apps.

## Introduction

### Background

A significant growth of research in well-being [1] and affective health [2] has taken place in the last decade across a range of disciplines, from human-computer interaction (HCI) and science and technology studies to clinical psychology and psychiatry. This range reflects the interdisciplinary work in this space, and we argue the unique position of the HCI discipline to articulate the design knowledge required for digital well-being interventions. Such work includes novel affective interfaces intended to support real-time awareness of emotions or their regulation [3-7], novel design approaches emphasizing the importance of the human body such as soma design [8], novel technologies supporting reflection and meaning making [9], and those intended to train meditation or mindfulness skills [10] or to conceptualize meaning [11]. Other strands of HCI work have focused on ill-health, such as mobile apps for cognitive behavioral therapy [12] and empirical studies exploring ways to support vulnerable users; such as those living with depression [13], dementia [14], addiction, or the compulsive use of technology; including screen time research [15,16]. Much of such work frames mental well-being as “positive emotional, psychological and social health” [17], whereas digital well-being is broadly seen as the result of being able to use technologies in productive and healthy ways without the negative consequences of dependency, distraction, or risks to users’ privacy [18].

A specific body of work has focused on interventions supporting smartphone nonuse, for instance, by increasing interaction cost to discourage smartphone app use [19]. Both limiting phone use and increasing interaction cost can be conceptualized within the slow movement, where technology is reframed with the aim of pausing and reflecting on its use [20]. Other examples of interventions of smartphone nonuse include apps such as MyTime to make users aware of their tracked use data, which in turn prompt them to reflect upon their use and especially the problematic use [21]. In addition, Roffarello and De Russis [22] argued that current digital well-being apps’ focus on self-monitoring may not be a sufficient mechanism to change users’ behavior with smartphones. Moreover, Roffarello and De Russis [22] also pointed out the limited exploration of the effectiveness and theoretical underpinning of digital well-being apps, whereas van Velthoven et al [23] highlighted also the insufficient investigation of the positive effects of regulating problematic smartphone use with digital interventions. The nascent research exploring the effectiveness of digital well-being apps has been limited, with only 1 study focusing on the analysis of users’ qualitative reviews of commercial apps [22]. Their findings indicate that such apps are liked, especially in studying, working, sleeping, parental control, and free time contexts, albeit limited in supporting behavior change and habit formation toward more conscious smartphone use.

In addition, the theoretical underpinning of digital well-being apps has also received limited attention. In this respect, most work has looked at their adoption [24,25], leveraging, for instance, technology acceptance theories [26,27], including the more recent technology acceptance life cycle model [28]; although these models are rather generic, they are leveraged for personal or domestic technologies. Scholars such as Douglas et al [25], Lukoff et al [29], Lyngs et al [30], Kim et al [31], or Colombo et al [3] have also identified other theories more relevant to digital well-being apps, such as the uses and gratification theory [19,31], theory of planned behavior [32], dual system theory [33], nudge theory [34], framework for behavior change [35], or theories for regulation [36]. However, it is less explored how such theories could actually inform the developing of commercial well-being apps.

Given the limited research on the theoretical and evidence-based aspects of digital well-being apps [23,25], we argue that unpacking the functionalities of the most used commercial apps is an important initial step toward better designing them. The exploration of functionalities and features of mobile apps is an emerging research area, with initial HCI work focusing on digital interventions and especially development of apps for specific conditions such as depressions [37,38] or for supporting, for instance, mindfulness [39,40] or physical activity [41]. In contrast, the functionalities of digital well-being apps have been less investigated. A noticeable exception is the exploration by Roffarello and De Russis [22] of 42 digital well-being apps and their descriptions on Google Play, whose findings indicate the following as key features: (1) tracking user behavior through phone unlocks, phone and app time, and app checking; (2) data presentation through phone and app summary, charts, daily or widget recap, and social comparison; (3) phone interventions through timers and blockers; (4) app interventions through timers, blockers, and notification blockers; and (5) extra features such as motivational quotes or rewards. However, given the brevity of apps’ descriptions available on marketplaces, a richer source to identify their key functionalities is the actual use of the apps, with authors, as HCI experts, adopting the role of the user by directly interacting with the apps—a method previously used for app reviews [38,42,43].

Specific functionalities of digital well-being apps have been also explored through research prototypes usually implementing tracking and notifications [44,45], whereas others included also specific interventions for limiting use [21,46]. For instance, the Socialize [22,47] app integrates the most common functionalities of tracking, data presentation, real-time notifications, and blocking use, which were evaluated in the wild with 38 young people over 3 weeks. Findings indicate improvements in terms of problematic use, measured through the phone addiction scale, and self-regulation, measured through the general self-efficacy scale. Although this is one of the few studies involving measures to explore the effectiveness of a digital well-being app, the Socialize app itself does not appear to be novel, borrowing common functionalities of commercial apps, whose theoretical grounding is limitedly unpacked. The Focus app [48] is another research prototype that leverages Nielsen’s heuristics to support tracking phone use and the blocking of any app, indeterminately or for a limited time set by the user, with the option to unblock them at any time, and provision of educational content on digital addiction. To mitigate overuse from a broader perspective, another research prototype, the FeelHabits app [49], tracks and notifies users about their use of specific apps; albeit rather than on a smartphone alone, this apps tracks use across devices and blocks them if limits set by the user are exceeded.

Another strand of scholarly work with richer theoretical underpinning has focused on restrictive and coercive interventions intended to be stronger than persuasive interventions by supporting users to commit to self-impose limits of use while the phone is blocked [31]. The framework for influencing behavior change [35] suggests the following four types of influence: persuasive (explicit and weak); coercive (explicit and strong); seductive (implicit and weak) and decisive (implicit and strong), which are based on the influencing force (strong and weak); and salience (explicit and implicit). Inspired by this framework, Kim et al [31] designed and evaluated GoalKeeper, a smartphone app featuring both a *weak lockout*, that is, the phone is locked increasingly longer (eg, 1, 5, 15, 30 and 60 minutes) each time the user exceeds the time they have previously set for use, with each lockout being mitigated by a temporary 15-minute allowance time, and a *strong lockout*, that is, the phone is locked until midnight without any allowance. Their findings indicate that both mechanisms were more effective than mere notifications of use, with the strong lockout being the most effective, as users set longer limits for not using their phones. Although in the latter case users experienced also more frustration, this was mitigated by the flexibility of setting their own limits and one-time opportunity to modify it.

### Objectives

Despite this growing academic interest in digital well-being, the commercial apps far outweigh the research prototypes in terms of uptake. Thus, the increased adoption of commercial well-being apps offers an opportunity to explore their potentially richer set of functionalities, and the aim of this paper is to articulate these functionalities as well as the novel design implications informed by them to better inspire the design of technologies for well-being. To address this aim, we focused on the following research questions:

1. What are the key functionalities of the top-rated digital well-being apps?
2. What theoretical underpinning supports these functionalities?
3. What design guidelines for digital well-being apps can be informed by these functionalities?

Our contributions are 3-fold. First, we unpacked richer insights about tracking and monitoring functionalities in terms of user profiling and understanding of monitoring as a *complete, location-based, and flexible practice* that can benefit from tailored, time-based visualizations. Second, we identified 4 interventions for limiting use including richer understanding of different types of obstacles for limiting use and specific features for less explored functionalities such as supporting awareness for reaching use limits, focused attention, and motivation to keep within set use limits. Third, grounded in our findings, we generated 6 design implications for digital well-being apps.

## Methods

### App Selection

To identify the digital well-being apps, in winter 2019, we searched for free apps in Google Play Store using the following search terms: *digital wellbeing*, *digital detox*, *detox apps*, *unplugging*, and *distraction*, which is a new direction given that extensive previous work on such apps has prioritized addiction and screen time [22]. We have focused on Google Play because its apps represent the largest global market share, >2.5 greater than iOS apps [50], whereas the latter is also more restrictive in terms of available information [22]. However, future work could extend this exploration to other platforms.

**Figure 1:**
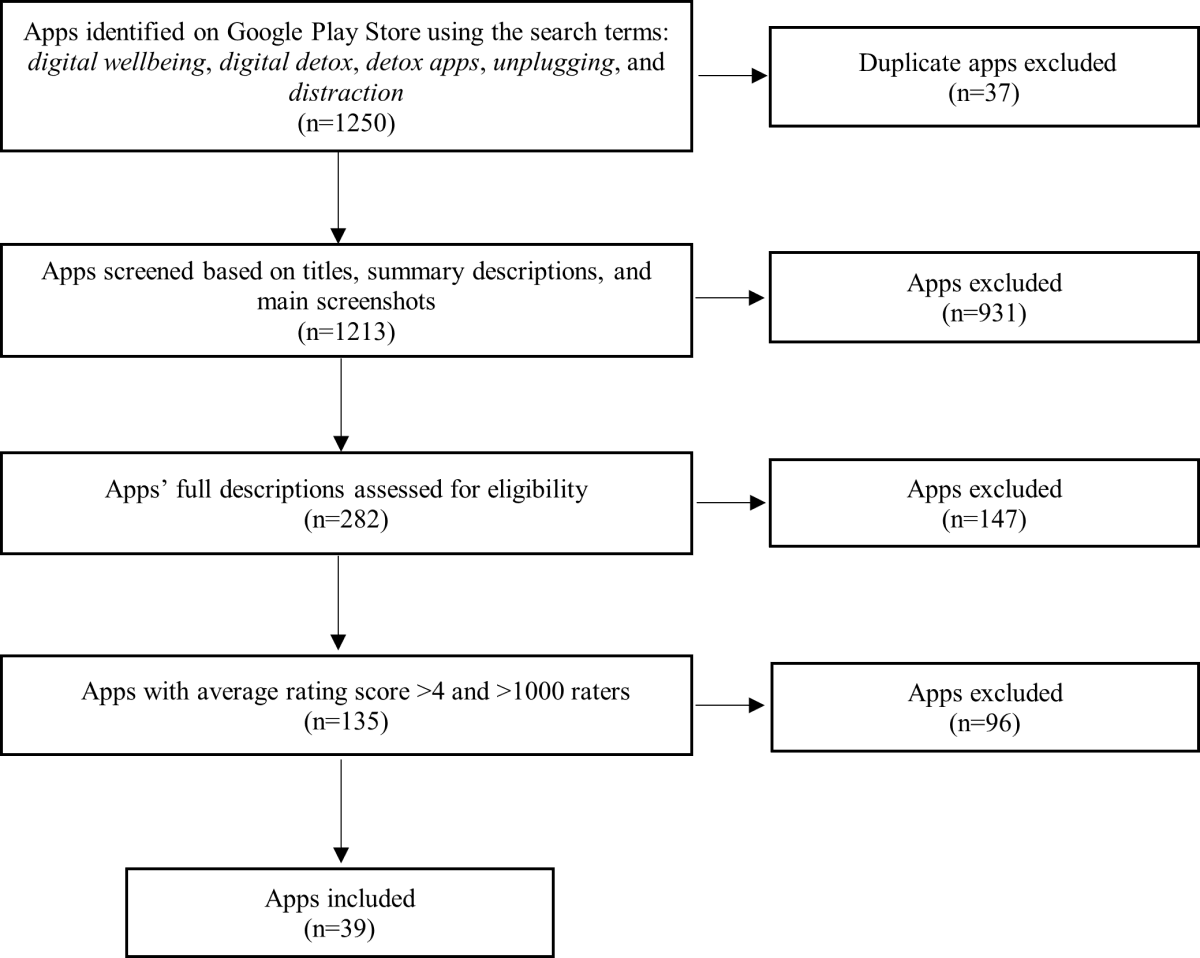
PRISMA (Preferred Reporting Items for Systematic Reviews and Meta-Analyses) diagram for the searching and screening of digital well-being apps.

For each search term, the top 250 most relevant apps returned on Google Play were retained, totaling 1250 apps, with 37 duplicates. At the screening stage, after reading their titles, summary descriptions, and main screenshots, we excluded 931 less relevant apps such as fitness, activity planner, or nondigital detox apps. The eligibility of the remaining 282 apps was assesses based on their full descriptions, with further 147 apps being excluded such as utility apps, games, and general well-being and meditation practice apps. From the remaining 135 apps, we further excluded those with less than 1000 raters and with average rating score <4, leading to 39 apps to be included in our review. The PRISMA (Preferred Reporting Items for Systematic Reviews and Meta-Analyses) diagram for the searching and screening process for digital well-being apps is shown in Figure 1. We also note that of our 39 apps, 12 (31%) are also available on the Apple Store, with 7 (58%) of them having user rating >4.2.

Our final set consisted of 39 digital well-being apps (Multimedia Appendix 1 [19,21,22,31,34,44-46,48,49,51-57]), which were analyzed through two complementary methods: first, a review of their functionalities based on their descriptions from Google Play and, second, an autoethnography with the authors (SA and CS), as HCI experts directly interacting with them in order to viscerally understand how these functionalities work and are experienced by potentially users in their daily lives. Such interactions were iterated, involving at least two sessions for each app, lasting for at least 30 minutes. For the autoethnography, we used a Samsung Galaxy Note 9 phone with an Android mobile operating system.

### App Analysis

The first author (SA) evaluated all 39 digital well-being apps, whereas the second author (CS) evaluated 21% (8/39) of the apps. Through the use of both methods, the authors (SA and CS) iteratively revised the coding scheme over several months, a process that has followed a hybrid approach. This integrated deductive codes, informed by prior work on functionalities [22] such as tracking, data presentation, and interventions (Table 1). The inductive coding was informed the distinction between tracking and monitoring, the revision of intervention functionalities such as tracking phone or app use by setting limits and of data presentation or visualization and its subcategories such as numerical and diagrammatic format through charts, round diagrams, metaphors, heat maps, or reports. Particularly important are the new functionalities capturing 4 interventions for limiting use.

To better contextualize our review in scholarly work, we subsequently extended the list of apps with 17 digital well-being apps designed in academia, which we found through search on Google Scholar using the following keywords: *digital wellbeing application* or *digital wellbeing app*. This search returned 42 papers, which after reading their abstracts, led to 17 papers describing such apps [3,8,12,18,26,32,37,49,51,58-65]. The remaining 25 papers do not included digital well-being apps and for this reason they were excluded. We have explored the functionalities of the apps described in the 17 papers by applying the above coding system to their description, as not all of them were available to download from app marketplaces. All the tables provided in Multimedia Appendices 1-7 [19,21,22,31,34,44-46,48,49,51-57] include information on both commercial and academic apps.

**Table 1:** The main codes and subcodes from the app analysis.

Functionality codes and subcodes		Definitions
**Tracking**		
	Recording phone or app use	The tracking functionality supports the recording of phone or app uses.
	Visualizing tracked use data	The tracking functionality supports the visualization of tracked data.
	Profiling users	The tracking functionality supports profiling users based on tracked data.
**Monitoring**		
	Setting time limits of phone and app use, their scope and place	The monitoring functionality provides use time limits or supports users to customize them in terms of scope and place.
	Visualizing monitored data	The monitoring functionality supports the visualization of monitored data against set time limits of use.
	Providing flexibility for limiting monitoring	The monitoring functionality supports flexibility for limiting monitoring through allowances to extend use beyond the set time limit, excluding apps from being monitored, or discontinuing the monitoring.
**Interventions for limiting use**		
	Creating obstacles to limit phone and app use	This intervention supports creating different types of obstacles to limit phone or app overuse.
	Supporting awareness of reaching the set use limits of phone and app use	This intervention supports users’ awareness of reaching the set use limits through different types of notifications varying in content and form.
	Supporting focused attention away from phones and apps	This intervention supports users’ focused attention on main task and away from habitual phone and app use through training or white noise.
	Supporting motivation to keep within limited use	This intervention supports motivation to keep within limited phone and app use through rewards and penalties, motivational quotes or education, and social motivation.

## Results

This section starts with a brief overview of the descriptive characteristics and ethical aspects of the 39 apps, continues with the identified main functionalities of top-rated digital well-being apps and how they compare with the apps developed in academia.

### Descriptive Characteristics of Digital Well-being Apps: Ethics

The descriptive characteristics captured by our analysis include app category, target users, scientific underpinning and evidence base, and cost. Findings indicate that the top-rated digital well-being apps belong to 6 categories, albeit feature predominantly in the Productivity category (27/39, 69% apps), followed by fewer apps in the Tools (4/39, 10%), Personalization (3/39, 8%), Health and fitness (2/39, 5%), Parenting (2/39, 5%), and Lifestyle (1/39, 3%) categories. Together with their main aim of limiting phone overuse, this is an interesting outcome that can be linked to the ethical principle *of nonmaleficence* [2] to protect users from the negative impact of phone overuse. These can also be aligned with the ethical principle of *beneficence*, particularly the predominant instrumental value of digital well-being apps supporting increased productivity rather than their eudemonic value for supporting meaningful goals [66]. Arguably, the latter would further strengthen their beneficence potential.

Another important outcome, which can potentially hinder their beneficence is the limited science base of digital well-being apps, with 97% (38/39) of the apps not specifying if they are backed up by research, the only exception being the Focus To-Do app described as a *science-based app*. This indicates the importance of these apps unpacking in their descriptions the scientific underpinnings informing their design and any available outcomes from evaluation studies. This, in turn, will support users make more informed choices regarding their beneficence.

The target users of digital well-being apps appear to be unrestricted, with most of them available to users of all ages, which reflects the principle of *justice*. Indeed, all but 4 apps are rated on Google Play as “PGEI 3,” which stands for “Pan European Game Information.” The remaining four apps do not specify any age: Brain Focus Productivity Timer, Lock Me Out, SleepTown, and Sma-Phospital. Interestingly, the design of the apps does not vary with age, as we see the same functionalities for both children and adults. We also examined whether the target users include the clinical population. Findings indicate that of the 39 apps, 38 (97%) do not specify clinical user groups, whereas 1 (3%) app, that is, AppBlock, mentions its suitability for children or adults with attention deficit hyperactivity disorder. This suggests that digital well-being apps predominantly target users without specific conditions or health concerns. However, given their value for supporting attention, some of these apps may be beneficial for users with attention deficit. Future work should further explore this.

Also related to justice, the cost of the digital well-being apps is an important aspect that can increase or limit diverse users accessing them. Regarding cost, an important outcome is that although all the 39 apps are free to download, only 11 (28%) are entirely free to use, whereas 28 (72%) offer in-app purchase mostly for removing adds, unlocking premium features, or subscribing to premium versions of the apps. This is an important outcome indicating that most functionalities of these apps are freely available, making their use particularly inclusive.

Digital well-being apps have an interesting relationship with the ethical principle of autonomy. On the one hand, these apps tend to limit one’s use of phone or apps; on the other hand, consistent findings have shown that autonomy is already impaired [67] when people live with some form of addiction such as phone overuse.

### Functionalities of Digital Well-being Apps

#### Overview

We now turn our attention to the key functionalities of digital well-being apps. The iterative analysis led to specific functionalities that can be broadly grouped into the following six main functionalities: tracking use of the phone or apps, monitoring use against set limits, and the four functionalities that highlight interventions for limiting use, namely, creating obstacles for the phone or app use, supporting awareness of reaching the set use limits, supporting focused attention, and support motivation to keep within limits of use. Each of these functionalities is further detailed.

#### Tracking Overall Phone and App Use: User Profiling

Findings indicate that 28 digital well-being apps automatically track or record overall phone use, use of specific apps, or both (Multimedia Appendix 2 [19,21,22,31,34,44-46,48,49,51-57]). In particular, (1) the overall use of the time spent on the phone was captured by 11% (3/28) of the apps through overall screen time across all apps measured per minute, hour, day, or week or the number of times the phone unlocks per hour or day; (2) the use of specific apps that provide users the choice to select them to capture only their screen time was captured by 54% (15/28) of the apps; and (3) 21% (6/28) of the apps tracked both the overall use of the phone and the use of specific apps. Other digital well-being apps provide users with the choice to select the time when the tracking can occur, for instance, between 9 AM and 5 PM, but not outside of the specified time window. Tracking can also be contextualized, with three apps (AppBlock, Instant-Quantified Self, and Lock Me Out), allowing its coupling with physical locations specified by users.

Regarding visualization, the tracked use data tends to be provided in numerical and diagrammatic format through reports (27/28, 96%), charts (21/28, 75%), round diagrams (9/28, 32%), metaphors (4/28, 14%), or heat maps (1/28, 4%; Multimedia Appendix 2). The four apps providing metaphoric visualizations are Forest: Stay focused, Focus To-Do: Pomodoro Timer & To Do List, SPACE, and SleepTown, with the latter’s visualization consisting of raising in-app towns when maintaining regular sleep hours. In addition, from the 27 of the apps including reports, 20 (74%) provided daily and weekly reports of screen time and 7 (26%) provided only daily such reports.

Findings also indicate that of the 39 apps, 10 (26%) extend the tracking functionality to also inform user profiling. Of these 10 apps, 4 (40%) use either the tracked data of app use (App Usage and Screen Time) to generate categories of used apps for broader purposes such as productivity and social, or ask users to identify these categories (SaveMyTime and Boosted). In addition, of the 10 apps, 3 (30%) provide users the option of creating different profiles for different settings that could be used to support different levels of limited use of the phone or apps, both with payment (HelpMeFocus) or without payment (Stay Focused), for instance, by allowing them to specify the location or specific Wi-Fi network where set limits are activated (AppBlock). This is important, as it indicates the flexibility of the interventions for limited use to the situatedness of users’ different contexts such as homes or work. Finally, the YourHour app also aims to identify levels of phone addiction based on tracked data, whereas the Digital Detox app offers predefined levels of limited use that users can choose from. These 2 apps are interesting, as they attempt diagnosis of smartphone addiction and prediction of the intervention intensity. Although smartphone addiction is not yet a clinical condition featured in the *Diagnostic and Statistical Manual of Mental Disorders*, its problematic behaviors as diagnostic criteria have started to be explored [68]. In addition, several scales have been developed for measuring phone addiction [65,69] that meet the psychometric properties of validity and reliability. If digital well-being apps aim to identify users’ level of addiction, which will allow for a better tailored intervention, these scales are useful to consider.

Interestingly, of the 39 apps, the remaining 11 (28%) that do not provide tracking functionalities include 8 (73%) apps supporting focused attention usually on offline activities (Forest, Boosted, Pomodoro Smart Timer, Brain Focus Timer, SleepTown, Engross, Visual Timer, and Hold), 2 (18%) launcher apps minimizing the number of apps being displayed (LessPhone Launcher and Before Launcher), and 1 (9%) app for turning off email notifications (Quite for Gmail).

#### Monitoring Phone and App Use Against Set Use Limits or Set Time Limits for Focused Attention

Apart from tracking, most digital well-being apps also allow setting use limits to track phone or app use against them (25/39, 64%); Multimedia Appendix 3 [19,21,22,31,34,44-46,48,49 ,51-57]). The distinction between tracking and monitoring is that monitoring is based on user intentions to self-limit their use, whereas tracking merely captures the time spent on apps or phone without any such limits. Thus, tracking becomes a prerequisite activity, performed first to explore one’s use patterns, and based on this information, use limits can be set. Indeed, all apps supporting monitoring also support tracking; however, 33% (13/39) of the apps, although supporting tracking, do not support monitoring. This is an important outcome as arguably, monitoring is better positioned to support behavior change toward limiting use than mere tracking; however, approximately 36% (14/39) of the top-rated apps do not support monitoring.

Whereas most apps (25/39, 64%) support setting limits for using the phone or its apps, the remaining 36% (14/39) of the apps include 8 (57%) apps that allow people to focus attention by setting time for offline activities and therefore away from phones and apps; 3 (21%) apps providing only the tracking functionality (Smarter Time, Sma-Phospital, and Usage Analyzer); 2 (14%) launcher apps minimizing the number of apps being displayed; and 1 (7%) app for turning off email notifications (Quite for Gmail). The prevalence of apps for focused attention on offline activities is an interesting and less explored monitoring aspect of digital well-being apps.

The monitoring functionality allows user setting of the scope and place of limited use, visualization of monitored content, and, interestingly, options for limiting monitoring. With respect to the scope of the limited use, more than half of the monitoring apps offer options to reduce the use of some of the installed apps (13/25, 52%). This means that while using these digital well-being apps, some apps’ use remains unmonitored. In contrast, the remaining digital well-being apps extend this option to monitor use to all apps on the user’s phone (6/25, 24%) or to the phone itself (7/25, 28%). Setting use limits can also be activated at specific locations, either specified through the phone GPS (3/25, 12% apps) or Wi-Fi network (1/25, 4% apps), although only a few apps offer these options.

Findings also indicate that 56% (22/39) of the digital well-being apps support a more forgiving or *flexible monitoring* by allowing users to limit their monitoring in 3 ways. This includes allowances to extend use beyond the set time limit (9/22, 41% apps) and the option to exclude specific apps from being monitored (19/22, 86% apps). Allowances are breaks during the set nonuse time limit so that users can continue to use the phone or the apps despite being during their set nonuse time limit, with or without (financial) penalties, although the number of breaks, and their duration is either capped or uncapped. This can also include terminating the nonuse time limit earlier than it is actually due (4/22, 18% apps). A total of 49% (19/39) of the digital well-being apps also offer the option of excluding specific apps from being monitored against time limits, especially apps such as App Usage–Manage/Track Usage, AntiSocial, and My Phone Time. In addition, 36% (14/39) of the apps allow users to discontinue monitoring when they reached the set use limit.

Regarding visualizations, monitoring function engulfs tracking one, so that it supports the visualization of tracked data. However, visualizations specific to the monitoring functionality are offered by less than half of the digital well-being apps (19/39, 49%). This is an important outcome, suggesting the value of considerably extending such visualizations within the monitoring functionality. These 19 apps provide monitoring-specific visualizations of (1) time unspent out of the use time limit, that is, count down (n=12, 63% apps), (2) time spent out of the use time limit (n=6, 32% apps), or (3) even time overspent as a percentage of the time limit (n=1, 5% apps). These are provided in either text form (12/19, 63% apps) and diagrammatic one as circles (4/19, 21% apps) or progress bars filled or unfilled gradually with colors (3/19, 16% apps) until the set time limit is reached. Interestingly, the monitoring of focused attention, usually during offline activities, can also be visualized, usually through time unspent out of the focus time (or time for not using the phones and apps), through countdown timers (3/19, 16% apps), or circle progressively unfilled with color (1/19, 5% apps).

#### Interventions for Limiting Use of Phones and Apps

Findings indicate four interventions for limiting the overall use of the phone or its installed apps, which include creating obstacles to limit use, supporting awareness of reaching the set limits, supporting focused attention, and supporting motivation for limiting use, which are further detailed.

#### Creating Obstacles to Limit Phone and App Use

The first intervention consists of creating obstacles for excessive phone or app use (21/39, 54% apps). Obstacles can be classified according to their force (strong or weak); saliency (explicit or implicit); temporal aspects such as being activated before, during, or after excessive use; and social aspects such as parental control or social commitment (Multimedia Appendix 4 [19,21,22,31,34,44-46,48,49,51-57]). Obstacles also differ with respect to their source (being generated by the digital well-being app or by users) and could be tailored to user profiles.

The identified strong obstacles feature predominantly in commercial apps (18/39, 46%). These obstacles that cannot be circumvented include the lockout of phones and apps beyond the set time limit of use (14/18, 78% apps), interrupting use while the set use time has been reached (12/18, 67% apps), and unchangeable time limits of phone and app use (6/18, 33% apps). In contrast, weak obstacles have features in much fewer apps (5/39, 13%), with only one app providing both strong and weak obstacles, that is, StayFree. Weak obstacles do not directly restrict use but make it more difficult through notifications from phones or apps after overuse (4/5, 80% apps), notifications inside the digital well-being app when reaching the time limit (4/5, 80% apps), and microboundary interactions that make it more difficult for users to access their apps targeted by limited use (2/5, 40% apps). Microboundary interactions are particularly interesting, as although theoretically explored in academic research, they have been limited, implemented through design. Such interactions feature in two apps (LessPhone Launcher and Before Launcher) and consist of *launchers* as substitute home screens for users’ phones that display only a reduced number of apps so that accessing other apps requires additional clicks for navigating from the launcher to them.

According to their saliency, most obstacles are explicit such as lockout (8/39, 21% apps), set time limits for phone and app use (14/39, 36% apps), and textual or visual notifications (4/39, 10% apps), whereas others are implicit such as launchers (2/39, 5% apps) or activation of the dimming mode of the phone’s screen when a set time limit was reached (1/39, 3% apps). This much lower number of implicit obstacles is interesting, suggesting a less explored design space and their potential value of complementing explicit obstacles.

With respect to the temporal aspect, most obstacles are created before the use of a phone or app and activated during the set limited time for using the phone or apps. The exception is flexible time limits, which can be changed not only during but also after the set time limit for use has ended.

The obstacles also have a social dimension, albeit only 13% (5/39) of the apps implemented them, in two forms: parental control (4/5, 80% apps) or social commitment (1/5, 20% apps). Regarding the latter, the Forest app leverages the feeling of failure to social commitment as a type of obstacle to prevent users from accessing apps while with friends.

Regarding the source, obstacles can be created by the digital well-being app or the user. The former leads to automatically generated obstacles, usually through user profiling (11/39, 28% apps), whereas the latter leads to customized obstacles (13/39, 33% apps). Apps allowing users to set use limits usually restrict this option to specific apps rather than all apps. Examples of the automatic setting of use limits feature in the YourHour app, which provides users short quizzes to identify if the app is used for work or entertainment. Another example is the SPACE app, supporting limited phone use through automatically suggested limits. Interestingly, two apps allow users to create multiple profiles, each profile with a particular setting to be assigned to different tasks (HelpMeFocus and Stay Focused). This is an interesting option, allowing users different modes of engaging with specific apps, which could, for instance, help with the context setting such as work or leisure, and different phone use for each.

Finally, different types of obstacles may be tailored to different user profiles for matching, for instance, the level of addition (1/39, 3% app) or users’ preference for a specific level of digital detox (namely, easy, medium, and hard) that are proposed to users to choose from (Digital Detox app). Interestingly, no apps attempt to recommend interventions at different levels (weak or strong) based on tracked data. This is a less explored feature with potential to provide adaptive interventions better tailored to users’ needs.

#### Supporting Awareness of Reaching the Set Use Limits of Phone and App Use

The second intervention is supporting awareness of reaching the set limits of use and is provided by 33% (13/39) of the apps (Multimedia Appendix 5 [19,21,22,31,34,44-46,48,49,51-57]). Such awareness is predominantly supported through explicit notifications of reaching the set time limits (12/13, 92% apps), usually in textual or diagrammatic form, with both push notifications that appear when the screen is both locked and unlocked, usually at the top in the status bar; (4/12, 33% apps) or pull notifications that appear suddenly in the middle of the screen as a small window alerting the user of something, sometimes these are big, covering most of the screen (7/12, 58% apps). Notifications can be provided both in the digital well-being apps about the use of the phone or its installed apps (13/39, 33% apps) and as embedded within a specific app when the time limit relates to that app (11/39, 28% apps). In contrast to explicit notifications, implicit ways to support awareness of reaching the time limit include screen dimming. Although less common (1/39, 3% apps), these are interesting, more subtle ways to notify users of reaching their use limits for specific apps or phones and to persuade disengagement. Although both notifications and screen dimming are provided in real time, daily reminders to review tracked data support a higher level of awareness beyond a specific instance of *in the moment* use and more about the historic user over the day (7/39, 18% apps).

#### Supporting Focused Attention on Primary Tasks and Away From Habitual Phone and App Use

The third intervention supports focused attention and features in >70% (29/39) of digital well-being apps (Multimedia Appendix 6 [19,21,22,31,34,44-46,48,49,51-57]). These include all apps that support monitoring (25/39, 64%) and four additional ones: Boosted, Pomodoro Smart Timers, Engross, and Hold. By aiming to limit phone and app overuse, digital well-being apps implementing the monitoring functionality implicitly support focused attention on the main task because they prevent the user’s attention from being hijacked by habitual phone and app use.

Findings also indicate that, of the 39 apps, 8 (21%; four that support monitoring and four that do not: Boosted, Pomodoro Smart Timers, Hold, and Engross) explicitly target the training of focused attention. These apps encourage users to stay away from their phone to focus on specific offline tasks for a set time. This use of a time limit is different from that in the monitoring functionality, as people are supported to practice the adaptive behavior of maintaining attention for a set time away from the phone rather than resisting for a set time the temptation to use the phone.

In addition, of these 8 apps for training focused attention, 5 (63%) also provide users with white noise to better facilitate concentration. This is an interesting outcome, and although these apps provide limited evidence for its value, scholarly work indicates that white noise, defined as “task-irrelevant auditory input containing many frequencies of equal intensities” [8], has potential to improve cognitive performance in both healthy adults [17] and those with attention deficit [70]. Mechanisms that could explain the benefits of white noise include its ability to moderate brain arousal by inducing neural noise, which at specific dopamine-based thresholds could stimulate cognitive performance [58].

#### Supporting Motivation to Keep Within Limited Use of Phone or Apps

The fourth intervention supports motivation for limiting phone and app use (12/39, 31% apps; Multimedia Appendix 7 [19,21,22,31,34,44-46,48,49,51-57]). Findings indicate 3 mechanisms for supporting motivation. The first is the reward and penalty feedback usually implemented by those apps that support monitoring (7/12, 58% apps), with rewards being provided when users successfully kept within their set use limits of their phones and apps. The main types of rewards leverage gamification principles and consist of badges at different levels (2/7, 29% apps), points (2/7, 29% apps), in-app coins (1/7, 14% apps), building in-app trees (Forest) or towns (SleepTown), or motivational quotes (4/7, 57% apps). The main categories of penalty content are metaphoric and consist of in-app tree withers (Forest) or town-building collapses (SleepTown). Interestingly, however, most monitoring apps (20/29, 69%) do not support such motivation through rewards and penalties.

Second, in addition to the reward and penalty feedback provided on the basis of successful or unsuccessful keeping within set limits of phone or app use, other types of motivation are provided to support behavior regulation of limiting use, both during and even before the actual behavior of limiting use. This less common type of motivation consists of motivational quotes, either provided by the app (two apps: Stay Focused and HelpMeFocus) number and names or generated by the user (two apps: StayFree and App Usage–Manage/Track Usage); educational content about phone and life balance (one app: SPACE); or motivational stories written by other users (one app: YourHour).

Third, social support is another form of motivation, whose role in facilitating behavior change has been much acknowledged [71]. An important outcome is the limited number of apps that encourage social support to limit phone or app use, through competition (5/39, 13% apps), collaboration (5/39, 13% apps), or both (3/39, 8% apps). This is distinct from the identified emphasis on competition [72]. For instance, the SPACE app allows comparing such progress of limited use. In contrast to this competition social motivator, our findings also show 13% (5/39) of the apps leveraging collaboration, in which family members, friends, or broader social networks are used. For instance, the SleepTown app allows sharing sleep time goals with friends and setting similar sleep goals with them. Another example is the Hold app, which provides different ways to share focus time through finding nearby Bluetooth-enabled devices to encourage focused attention in the group. The Hold app also integrates collaborative and competitive aspects, for instance, by ranking users according to the points they gained from their time spent on focusing tasks, most often offline ones. Apps leveraging competition can also integrate social recognition. For example, the Hold app rewards the top-ranked users according to their points with a crown icon next to their username, and the Focus app rewards the first three users with a trophy icon next to their usernames: gold, silver, and bronze.

### Comparison of Commercial Digital Well-being Apps With Academic Ones

This section focuses on the comparison of the functionalities of the apps developed in academia with those of commercial apps, with a specific focus on how they differ. It is not surprising that most academic apps share the tracking and monitoring functionalities available in commercial apps. For example, the lockout mechanism that blocks the phone until midnight when reaching the use limit [31] is similar to blocking apps and phone when the user exceeds the defined time limit in some commercial apps (ie, Ubhind). Similarly, blocking and scheduling blocking in the academic app Forest [48] are comparable with those in the commercial app AppBlock. An interesting distinction concerning tracking and monitoring is the new form of visualization of tracked data in academic apps, namely, timelines.

In terms of interventions for limiting use, findings indicate additional key distinctions between commercial and academic apps for digital well-being. Regarding creating obstacles to limit phone or app use, important distinctions concern the force and saliency of the created obstacles and their temporal aspect and source. With respect to force, commercial apps predominantly use strong obstacles such as phone or app blocks (14/39, 35% apps) instead of weak obstacles such as notifications or microboundary interactions (5/39, 13% apps), with only 3% (1/39) of the apps providing both strong and weak obstacles. In contrast, academic apps take a more balanced approach, using equally both strong (10/17, 59%) and weak (11/17, 65%) obstacles, with 29% (5/17) of these apps using both strong and weak obstacles. Given the nascent research exploring the effectiveness of digital well-being apps, academic work is more likely to use both types of obstacles to compare their effectiveness.

With respect to the saliency of obstacles, almost half of the commercial apps (17/39, 44%) specify saliency, with all but 1 featuring explicit obstacles (which also tend to be strong), whereas the SPACE app features implicit obstacles. In contrast, almost all academic apps (16/17, 94%) involve explicit obstacles, that is, mostly notifications. What is interesting here is the innovative use in academic apps of a new type of obstacles for restricting use through design frictions. These could involve mandatory cognitive tasks such as entering several digits as users attempt to start interacting with apps targeted for limited use [57] or entering 30- or 10-digits try [19], which, when compared with merely pressing OK, indicates that the more complex the cognitive task, the more likely that users will restrain from engaging with those apps. Commercial apps present limited such cognitive tasks, with one exception being the MMGuardian app, which requires entering a password by parents to prevent the child from removing the app or modifying the set time limit of use.

Findings also indicate differences regarding the temporal aspects of obstacles to use. Although commercial apps use these obstacles predominantly after use of the targeted apps (15/39, 38%), as opposed to during use (4/39, 10%), academic apps take a more balanced approach using such obstacles equally during (8/17, 47%) and after the use of targeted apps (8/17, 47%), with 12% (2/17) of the apps using them both during and after use. This suggests not only the value of providing flexibility and users’ choice but also the importance of real-time obstacles in limiting phone or app overuse in real time.

Regarding obstacles’ source, commercial apps use mostly obstacles set and customized by users (15/39, 38%) rather than obstacles set automatically (6/39, 15%); in contrast, academic apps feature more automatically set obstacles (10/17, 59%) than those set by users (6/17, 35%).

Scholarly work on digital well-being apps has also focused on the types of apps that users are more willing to limit use. In this respect, empirical findings indicate that users were willing to restrict the use of specific apps such as messaging ones [56], as well as social media or games apps [21]. Academic work has also explored limited use not only beyond individual devices such as phones but also across multi-devices and their context of use [46,49]. Similar work has looked, for instance, at chatbots to notify users of their smartphone use [51] or video platforms supporting preschoolers to self-manage their phone and app consumption [52].

The second intervention, intended to increase users’ awareness of reaching their limits of phone or app use, also shows differences. Although both sets of apps use mostly explicit notifications to support such awareness, academic apps do so more (8/17, 47%) than commercial apps (11/39, 28%). Interestingly, both sets of apps also used implicit notifications such as screen dimming featuring in the SPACE app and vibrations for notifying users when they exceeded their set time limit for phone use featuring in the Good Vibrations app [34].

The intervention targeting focused attention has been supported by both sets of apps through training for focused attention, with 21% (8/39) of commercial apps and 29% (5/17) of academic apps providing such training. Interestingly, commercial apps also feature white noise as a specific mechanism for supporting focused attention, whose effectiveness as part of digital well-being apps has been less explored, although a body of scholarly work has shown its value for relaxation [17,70].

Finally, the fourth intervention for supporting motivation to keep within set limits shows similar findings for the 2 sets of apps, with emphasis on rewarding user behavior when the goal of keeping within limits has been reached (9/39, 23% commercial apps; 3/17, 18% academic apps), albeit commercial apps show more diverse forms of rewarding content, usually leveraging gamification principles, as opposed to academic apps, which use merely points. In contrast, findings show much fewer apps leveraging punitive feedback when users fail to keep within set use limits for both commercial apps (4/39, 10%) and academic apps (1/17, 6%). In terms of social support, a small number of apps provide it to support cooperation (5/39, 13% commercial apps; 2/17, 12% academic apps), competition, and recognition (5/39, 13% commercial apps; 3/17, 18% academic apps).

Also unique to research on academic apps for digital well-being is the extended focus of their audience to include not only individual users as commercial apps but also groups of users. For example, such academic apps focused on enhancing self-regulation through groups of users collaborating or competing toward limiting their collective use of phones and apps [45,54], through limiting use as a family activity [45], or through providing in-app spaces for college students to restrict their phone use during class time [44].

## Discussion

### Principal Findings

We now revisit the research questions advanced in the *Introduction* section and articulate the novelty of our key findings. The first 2 research questions focused on identifying the key functionalities of the top-rated digital well-being apps and their theoretical underpinning. Our review of top-rated digital well-being apps indicates the following six main functionalities: tracking use; monitoring use against set limits; and four interventions for limiting use, namely, creating obstacles to limit use, supporting awareness of reaching the set limits, supporting focused attention, and supporting motivation for limiting use. In this section, we also theoretically position these functionalities and leverage them to articulate new implications for better designing digital well-being apps.

Findings indicate that >70% (29/39) of digital well-being apps provide tracking of use of phone or app data, visualized mostly through reports and charts. More than a third (11/29) of the apps providing the tracking functionality also support user profiling, either automatically from tracked data or through users’ entered data. This aspect of tracking has been limitedly explored in previous work [22,23]. Another key finding is that almost 30% (11/39, 28%) of the digital well-being apps do not support tracking phone or app use but support instead focused attention or tracking of offline activities. This is a key outcome with important design implications that we will revisit later.

The second functionality is monitoring phone or app use against set time limits, which is key for limiting their use. This functionality features in 64% (25/39) of our reviewed commercial apps. Interestingly, however, the remaining almost 35% (14/39) of the digital well-being apps do not support this functionality directly, albeit they monitor the time spent on offline activities, away from the phone and its apps. From the former set of apps monitoring phone and app use, most tend to target some of the apps installed on the phone, with fewer digital well-being apps monitoring the use limits of all the apps. An important implication here is designing for *complete monitoring* of all the apps installed on the phone and providing users with the choice of selecting the ones to monitor, as well as *location-based monitoring* currently limitedly supported, albeit useful for situating the monitoring behavior in a spatiotemporal context. We also suggest supporting *flexible monitoring* allowing circumventing the set use time limit, which can support ongoing motivation for monitoring phone consumption and regulating phone overuse behavior. Findings also indicate interesting time-based, monitoring-specific visualizations featuring in approximately half (20/39) of our reviewed apps, which are useful to be extended to all digital well-being apps.

With respect to the first intervention, almost half (16/39) of our reviewed commercial apps implement strong and explicit obstacles, such as blocking to limit phone or app use, with much fewer apps featuring weak or implicit obstacles, usually in the form of notification. Even fewer apps attempt to implement microboundary interactions using launchers as substitute home screens. Such obstacles can be generated either automatically or by the users, with only few apps tailing them to user profiles and none mapping the force of obstacles (strong or weak) to such profiles. These approaches suggest the value of using both sources, so that digital well-being apps could benefit from the customization of users’ set obstacles and potentially even more so from extending the use of automatically set obstacles. Although previous work suggested that strong obstacles, despite inducing frustration, can be preferred by users and are likely to be more effective than the less restrictive obstacles [22,46], the value of providing both strong and weak obstacles can be further explored, in terms of both effectiveness and user experience for more sustained and long-term change of one’s relation with their mobile phones. Our findings from academic apps also highlighted new explicit obstacles for restricting use through design frictions such as cognitive tasks, which, unfortunately, have been limitedly explored. However, these innovative obstacles open up an interesting design space, as frictions support users to pause before compulsively re-engaging with their phones and apps, and thus a more mindful interaction.

From the 25 apps that support monitoring, 13 (52%) support users becoming aware when they reach the set use limits of their phone or apps, mostly through explicit notification and much less through implicit ones such as screen dimming, whereas daily reminders support a high-level awareness of use patterns exceeding set limits. Academic apps also started to explore implicit notification, albeit in tactile modality, through vibrations. These implicit notifications open up a less explored design space for this intervention. Arguably, vibration-based notifications are weak obstacles, and illustrations of how nudge theory [34] can be leveraged in the design of digital well-being apps. Implicit notifications may be less intrusive and therefore more persuasive, although future work is needed to explore their specific benefits when compared with explicit ones.

An important outcome is the 2 ways of supported focused attention that digital well-being apps implement: The first is implicit support through the monitoring and limiting of phone or app overuse, and the second is the explicit training of attention by focusing on offline activities without phone use, including also exposure to white noise to support concentration, which has strong research underpinning [17,70].

A key functionality, less explored in previous research on digital well-being, is supporting motivation for keeping within limited use. For this functionality, we identified the following three mechanisms: reward and penalty leveraging gamification principles, educational and motivational content, and social support provided, however, by a limited number of apps and where cooperation among users is limited.

The theoretical underpinning of digital well-being apps has received limited attention. However, Roffarello and De Russis [22] suggested the value of grounding the design of well-being apps to support behavior change, habit formation, and self-regulation. As shown in the *Introduction* section, scholars have identified a range of theories that may inform the design of digital well-being apps, such as those of uses and gratification [19,31], planned behavior [32], dual system [33], nudge [34], framework for behavior change [35], or regulation [36]. However, it is less explored how such theories have been actually informing the developing of commercial well-being apps. However, the operationalization of these theories in this respect has been limited. In this section, we argue for the value of self-regulation theories.

Previous work has shown that tracking is a key functionality of digital well-being apps that captures the use of the phone and its apps [22]. However, this does not make the important distinction between the digital well-being app running in the background to collect such information and the user’s active effort to minimize phone use. The former is usually important in the early stage of digital detox when people want to understand their consumption patterns, whereas the latter follows with setting up limits to phone or app use. For this, we called the former tracking, and the latter monitoring, which is a better term for capturing or tracking data against a specific target. Most behavior-changing apps use monitoring toward specific goals such as exercising ones [41]; therefore, the link between monitoring and goal setting is crucial. We note the important alignment of monitoring functionality to the three ingredients of self-regulation as reflected in self-regulation theories: setting target standards, monitoring the current state against these targets, and activating processes to reduce any identified distance between the current state and the targets [59]. Thus, we argue that designing for monitoring functionality can benefit from theoretical grounding in self-regulation theories.

Regarding the intervention of creating obstacles to limit use, we have seen the value of both strong restrictive mechanisms and weak ones, mostly explored in academic research rather than reflected in commercial apps. We argue that weak and particularly implicit obstacles are illustrations of nudges, which nudge theory describes as persuasive attempts for behavior change that do not limit users’ choices [73,74]. Future work is needed to understand how nudge theory can be sensitively leveraged to rigorously inform such obstacles to use.

The intervention for supporting focused attention is particularly interesting, as it marks a shift away from limiting excessive use toward more mindful activities, either technologically mediated or offline, whose valuable side effect is limited use of the phone or apps. Rather than steering away from undesirable behavior, this intervention encourages engagement in meaningful and ideally desirable activities, subsequently supporting the most powerful appetitive rather than aversive motivation. We also highlight in this context the value of supporting users to understand and support their meaningful goals [66], which subsequently can address the phone overuse and the boredom often associated with it. However, goal theories have been limitedly discussed in relation to digital well-being apps.

The final intervention focuses on supporting motivation to keep within use limits. Although limitedly mentioned in relation to digital well-being apps, we suggest the value of broaden and build theory [75], where positive emotions are leveraged for increased self-awareness and behavior change. Illustrations of how this theory may be underpinning some of the identified functionalities include the provision of allowances for overruling the set use limits during monitoring. This is important for instrumental reasons both allowing the completion of some immediate tasks, and maintaining motivation in case of setbacks in meeting the set limits. In turn, this could broaden users’ resilience and more flexibly support the acknowledged high demands of self-regulation [36]. Future work is needed to explore effective ways for managing the negative emotions associated with setbacks.

### Design Implications for Digital Well-being Apps

#### Overview

The third research question focused on the design guidelines for digital well-being apps informed by our identified functionalities. For this, we articulate 6 implications for designing digital well-being apps including calling to move beyond screen time and support the broader focus of digital well-being; supporting meaningful use rather than limiting meaningless use; leveraging (digital) navigation in design for friction; supporting collaborative interaction phone overuse; supporting explicit, time-based visualizations for monitoring functionality; and supporting the ethical design of digital well-being apps. These implications open up a larger design space for digital well-being apps, going beyond the main tracking and monitoring functionalities [19,34,57].

#### Beyond Screen Time: Broader Focus of Digital Well-being

Although most of these functionalities focus on limiting screen time, echoing previous findings on addiction and phone overuse [22], an important outcome is that about a third (13/39) of our apps support focus of attention either by limiting distractions or by supporting focused attention often on offline activities, including training of attention. We argue that this bias toward screen time fails to reflect the larger body of HCI research on well-being that can inspire novel apps that may better support users’ skills for more mindful use of technologies. We call for stronger engagement of HCI research in the design of digital well-being apps that addresses this limitation. Indeed, our findings could mark a shift away from addressing a problematic behavior by explicitly limiting it but rather by supporting a high-level function that can arguably better address the root of the problematic behavior. There is an extensive body of work on mitigating the impact of interruptions [62,63] and a growing interest in mindfulness technologies [39,40,64,76] that can support the design of these apps for digital well-being aiming to support focus of attention.

#### Supporting Meaningful Use Versus Limiting Meaningless Use

Findings also indicate an important limitation of digital well-being apps reviewed in this work and in particular their rather narrow view of limiting use. We argue that this overlooks the broader goals for using technology in the first place and users’ different avoidance or approach motivations. For this, we can leverage goal theories and the distinction between hedonic and eudemonic or meaningful goals [66] and how the latter can be purposefully designed for. Emphasizing the meaningful use of technology [11] may be a better approach to avoid meaningless or habitual use leading to phone overuse, while accounting also for the scarcity of attention [77].

#### Leveraging (Digital) Navigation in Design for Friction

Findings highlight obstacles for preventing app use that can inform the design for friction [78] as a mechanism for slowing down interaction (such as information sessions at the start of using a mediation app), which we know little about. Our findings suggest harnessing the digital distance and navigation to the target app. This is supported by findings showing that navigation in the folder hierarchy and in the real world share the same neural correlates [61]. One can imagine that information architecture imposing additional digital navigation cost for reaching apps located deeper in the phone’s information hierarchy, whose use is to be limited, may mitigate against their overuse. We can also think of leveraging physical navigation, for instance, by allowing access to some apps only in physical locations that the user has to purposefully travel to, supporting thus fitness goals. Kim et al [31] positioned their app and this family of restrictive and coercive interventions within the HCI work on uncomfortable interactions aimed at helping people toward important goals while tolerating discomfort [60] and on design frictions through microboundaries consisting of small barriers enforced before an interaction to prevent habitual phone use [57].

#### Supporting Collaborative Interaction for Limiting Phone and App Overuse

Much work has shown the value of social support for behavior change, and our findings confirm that this is also an important intervention for digital well-being apps. Our outcomes echo previous ones showing the benefit of social support for limiting smartphone use, albeit by leveraging competition. We argue that the value of cooperation can be better harnessed in the design of digital well-being apps, both for limiting overuse and for training focus of attention. Our findings indicate that only 16% (9/56) of the apps in our app review implement social support as a built-in feature. This supports the argument presented in a study by Czerwinski et al [62] that social support is a feature needed in digital well-being apps, as current apps do not seem to leverage social support as a mechanism to enhance self-regulation.

#### Supporting Time-Based, Explicit Visualizations Tailored to Monitoring Functionality

In terms of data visualization, findings indicate a richer range of formats available for the monitoring of phone or app use against set limits compared with their mere tracking. This makes sense because tracking aims primarily to support users’ exploration and understanding, whereas monitoring aims mostly to support behavior change toward set goals [79,80]. Hence, although more ambiguous representations are useful to motivate and engage users during tracking, for the monitoring functionality, more specific formats and particularly those including timelines are more useful. However, we have seen that academic apps leverage timeline representations, whereas commercial apps do so to a lesser extent. The latter allow people to easily match on the timeline their behavior with the recorded data to not only understand the data but also use it for reaching the goals. These outcomes align with previous work on the value of the ambiguity of different types of captured data [4] to support users’ engagement in understanding it, particularly relevant in the tracking stage. In contrast, although the rationale of timeline visualizations has been limitedly unpacked in scholarly work, it can be grounded in the growing HCI interest in temporality [24] and its value for reflection, both in and on action [81]. Future work can compare the value of different visualization forms for supporting such reflection on data.

#### Supporting Ethical Design of Digital Well-being Apps

Despite their potential for supporting users with their phone overuse, most digital well-being apps have limited scientific underpinning and evidence base. They tend to target users without health conditions and tend to be inclusive, as many of their functionalities appear to be free. However, we call for extending the efforts toward a more research-informed and evidence-based design of digital well-being apps. This is particularly important because their beneficence can be limited by the risk of harming users with mental health conditions, as well as those who experience phone addiction. Such recommendation can be addressed to app market places or policy makers for regulating the requirements for their research underpinning. The most ethical challenge pertaining to these apps is supporting autonomy of users experiencing smartphone addiction [67]. However, given the challenges of diagnosing phone addiction, increased ethical sensitivity is required in this respect. In addition, more work is needed to explore how the shift toward increased autonomy can be best supported and by what features of digital well-being apps.

### Limitations and Future Work

We focused on Google Play, which limited our review of iOS apps not available on Google Play. Future work could extend this exploration to other platforms. Future work can also aim to further strengthen the scientific underpinning of the design principles of digital well-being apps, in terms of both their theoretical framing and evidence-based evaluation studies. Our findings indicate that despite the growing number of digital well-being apps, parts of their design space have been less explored, such as supporting awareness for reaching use limits, motivation to keep within set use limits, implicit obstacles rather than explicit ones, recommended interventions to determine the right type of obstacles according to the tracked data, and mechanisms for supporting focused attention. We encourage researchers and developers to focus on these aspects, and together with the key features identified in our study, they can significantly improve the design of digital well-being apps.

### Conclusions

We report on a functionality review of 39 commercial and 17 academic digital well-being apps. Findings provide richer understanding of tracking and particularly monitoring functionalities, together with 4 interventions for limiting use. These provide new understanding of the different types of obstacles for limiting use, as well as of specific features for less explored functionalities such as supporting awareness for reaching use limits, focused attention, and motivation to keep within set use limits. We conclude with 6 design implications for digital well-being apps, namely, calling to move beyond screen time and support the broader focus of digital well-being; supporting meaningful use rather than limiting meaningless use; leveraging (digital) navigation in design for friction; supporting collaborative interaction to limit phone overuse; supporting explicit, time-based visualizations for monitoring functionality; and supporting ethical design of digital well-being apps.

## Appendix

Multimedia Appendix 1 The reviewed top-rated digital well-being apps and academic apps, their user rating scores from 1 to 5, and their numbers of raters.

Multimedia Appendix 2 Tracking functionality for phone and app use, format for visualizing the tracked data, and user profiling based on tracked data.

Multimedia Appendix 3 Monitoring functionality: setting use or focus time limits, scope and place of limited use, visualizing time limit, and flexibility through 3 options (use allowance beyond time limit, exclude apps from time limit, and discontinuing tracking when limit was reached).

Multimedia Appendix 4 Interventions for limiting use: creating obstacles for limiting use differing in force, saliency, temporality, sociality, user profile, and source.

Multimedia Appendix 5 Interventions for limiting use: supporting awareness for reaching the set limit of use through different notification types, screen diming, and daily reminders.

Multimedia Appendix 6 Interventions for limiting use: supporting focused attention through training or white noise.

Multimedia Appendix 7 Interventions for limiting use: supporting motivation to keep within limited use involving different types and content, as well as social support of different types involving cooperation, competition and associated social recognition.

## Abbreviations

HCI: human-computer interaction
PRISMA: Preferred Reporting Items for Systematic Reviews and Meta-Analyses
