# Supplementary material for: Toward Research-Informed Design Implications for Interventions Limiting Smartphone Use: Functionalities Review of Digital Well-being Apps
Source: JMIR Form Res. 2022 Apr 19;6(4):e31730. doi: 10.2196/31730 (PMC9066336; doi:10.2196/31730)
Supplement: Multimedia Appendix 1 [file formative_v6i4e31730_app1.docx]

| **App ID** | **App name** | **Rating**  **score** | **Number of**  **app raters** |
| --- | --- | --- | --- |
| **Commercial apps** | | | |
| **1** | Google Family Link for parents | 4.6 | 347376 |
| **2** | Forest: Stay focused | 4.7 | 162902 |
| **3** | Parental Control - Screen Time & Location Tracker | 4.1 | 40983 |
| **4** | YourHour - Phone Addiction Tracker & Controller | 4.6 | 37483 |
| **5** | Focus To-Do: Pomodoro Timer & To Do List | 4.7 | 37278 |
| **6** | UBhind: No.1 Mobile Life Tracker/Addiction Manager | 4 | 33358 |
| **7** | SPACE: Break phone addiction, stay focused | 4.3 | 27606 |
| **8** | StayFree - Phone Usage Tracker & Overuse Reminder | 4.6 | 26437 |
| **9** | AppBlock - Stay Focused (Block Websites & Apps) | 4.5 | 24385 |
| **10** | Stay Focused - App Block & Website Block | 4.4 | 21389 |
| **11** | MMGuardian Parental Control App For Parent Phone | 4.1 | 16909 |
| **12** | Screen Time - Restrain yourself & parent control | 4.7 | 16887 |
| **13** | SaveMyTime - Time Tracker | 4.5 | 8968 |
| **14** | Detox Procrastination Blocker: Digital Detox | 4.3 | 7609 |
| **15** | Boosted - Productivity & Time Tracker | 4.7 | 7116 |
| **16** | AntiSocial: phone addiction | 4.2 | 6933 |
| **17** | App Usage - Manage/Track Usage | 4.3 | 6720 |
| **18** | Smarter Time - Time Management - Productivity | 4.3 | 5619 |
| **19** | ActionDash: Digital Wellbeing & Screen Time helper | 4 | 4972 |
| **20** | Digital Detox: Focus and fight phone addiction | 4.5 | 4403 |
| **21** | Keep Me Out | 4.1 | 4048 |
| **22** | Block Apps - Productivity & Digital Wellbeing | 4 | 3728 |
| **23** | Instant - Quantified Self, Track Digital Wellbeing | 4 | 3367 |
| **24** | LessPhone - The Original Distraction Free Launcher | 4.3 | 2696 |
| **25** | Minimalist launcher for focus \| Before Launcher | 4.4 | 2186 |
| **26** | Focus - Be Productive! | 4.5 | 2152 |
| **27** | My Phone Time - App usage tracking - Focus enabler | 4.4 | 2002 |
| **28** | Usage Analyzer: Apps, Data & History | 4.5 | 1685 |
| **29** | Pomodoro Smart Timer - A Productivity Timer App | 4.6 | 1287 |
| **30** | Screen Time & Parental Control App by ZenScreen | 4.1 | 1163 |
| **31** | Brain Focus Productivity Timer | 4.5 | 8947 |
| **32** | SleepTown | 4.4 | 6921 |
| **33** | Engross: Focus Timer, To-Do List & Day Planner | 4.4 | 5027 |
| **34** | Visual Timer - Countdown | 4.7 | 4702 |
| **35** | Lock Me Out: Freedom from phone addiction | 4.3 | 2920 |
| **36** | HelpMeFocus - Block Apps, Stay Focused. | 4 | 2873 |
| **37** | Hold - make it happen | 4.5 | 2386 |
| **38** | Sma-Phospital | 4 | 2203 |
| **39** | Quiet for Gmail | 4.7 | 1478 |
| **Academic apps including references** | | | |
| **1** | Focus [57] | None | None |
| **2** | Socialize [61] | Not available | Not available |
| **3** | Toringo [1] | Not available | Not available |
| **4** | FeelHabits [9] | Not available | Not available |
| **5** | Coco’s Videos [24] | Not available | Not available |
| **6** | MyTime [25] | Not available | Not available |
| **7** | Good Vibrations [54] | Not available | Not available |
| **8** | Let’s FOCUS [29] | Not available | Not available |
| **9** | PomodoLock [30] | Not available | Not available |
| **10** | Interaction restraint [55] | Not available | Not available |
| **11** | GoalKeeper [31] | Not available | Not available |
| **12** | LocknType [32] | Not available | Not available |
| **13** | AppDetox [43] | 4.4 | 3000 |
| **14** | Lock n’ LoL [35] | Not available | Not available |
| **15** | FamiLync [34] | Not available | Not available |
| **16** | NUGO [36] | Not available | Not available |
| **17** | The SAMS [39] | Not available | Not available |

The reviewed top rated digital wellbeing apps and academic apps, their user rating scores from 1 to 5, and their numbers of raters.
