# Supplementary material for: Toward Research-Informed Design Implications for Interventions Limiting Smartphone Use: Functionalities Review of Digital Well-being Apps
Source: JMIR Form Res. 2022 Apr 19;6(4):e31730. doi: 10.2196/31730 (PMC9066336; doi:10.2196/31730)
Supplement: Multimedia Appendix 2 [file formative_v6i4e31730_app2.docx]

| App ID | Tracking  phone use | Tracking  apps use | Visualizing tracked  data - formats | Profiling  users |
| --- | --- | --- | --- | --- |
| Commercial apps | | | | |
| 1 | None | Yes | Charts/Reports | None |
| 2 | None | None | Metaphors | None |
| 3 | None | Yes | Charts/Reports | None |
| 4 | None | Yes | Charts/Round diagrams/Reports | The app defines levels of phone addiction based on tracked data: addicted, obsessed, dependent, habitual, achiever and champion.  The app categorizes some used apps as productivity apps (and not considered in addiction level) |
| 5 | None | Yes | Metaphors/Reports | None |
| 6 | Yes | Yes | Charts/Reports | None |
| 7 | None | Yes | Metaphors/Round diagrams/Charts/Reports | Usage patterns are extracted from user quiz |
| 8 | None | Yes | Round diagrams/ Charts/Reports | None |
| 9 | None | Yes | None | User can create different profiles for limiting use based on time, location, Wi-Fi, usage limit, or launch count |
| 10 | None | Yes | Charts/Reports | User can create different profiles with different settings, i.e., daily usage limit. |
| 11 | None | Yes | Charts/Reports | None |
| 12 | None | Yes | Charts/Reports | The app categorizes used apps based on tracked data, i.e., social, entertainment, tools |
| 13 | Yes | None | Reports | Users can create goals to limit use time or to maximize valuable time. |
| 14 | Yes | None | None | None |
| 15 | None | None | Round diagrams/ Charts/Reports for tracked offline activities | Users can set time to increase offline activities and track them. |
| 16 | None | Yes | Charts/Reports | None |
| 17 | None | Yes | Heatmap/Round diagrams/ Charts/Reports | The app categorizes used apps based on tracked data, i.e., social, news, productivity. |
| 18 | None | Yes | Charts/Reports | None |
| 19 | None | Yes | Round diagrams/ Charts/Reports | None |
| 20 | Yes | None | Charts | The app provides predefined levels of digital detox, i.e., easy, medium, hard, grand master |
| 21 | Yes | None | None | None |
| 22 | None | Yes | Charts/Reports | Users can add restrictions based on daily or week limits and launch count limit |
| 23 | None | Yes | Reports | None |
| 24 | None | None | None | None |
| 25 | None | None | None | None |
| 26 | Yes | None | Round diagrams | None |
| 27 | None | Yes | Charts/Reports | None |
| 28 | None | Yes | Round diagrams/ Charts/Reports | None |
| 29 | None | None | None | None |
| 30 | None | Yes | Round diagrams/ Reports | None |
| 31 | None | None | Charts/Reports | None |
| 32 | None | None | Metaphors/Charts for tracked offline activities | None |
| 33 | None | None | Reports for tracked offline activities | None |
| 34 | None | None | None | None |
| 35 | Yes | Yes | None | User can create predefined rules to lock phone on specific locations, times of day, total screen time. |
| 36 | None | Yes | None | Users can pay to create different profiles with different settings, i.e., work time |
| 37 | None | None | None | None |
| 38 | None | Yes | Charts/Reports for tracked activities | None |
| 39 | None | None | None | None |
| Academic apps | | | | |
| 1 | None | Yes | Charts | None |
| 2 | Yes | Yes | Charts, Daily/Widget Recap | None |
| 3 | Yes | None | Floating widget | Users specify the total time screen |
| 4 | Yes | Yes | None | Users can choose temporal context restriction: “working days”, “holidays”, “morning”, “afternoon”, “night” and from 3 categories of screen time: multi-device apps, smartphone while using PC, multi device. |
| 5 | None | None | None | None |
| 6 | None | Yes | None | User can set daily time limit for the tracked apps |
| 7 | None | Yes | None | None |
| 8 | Yes | Yes | Timeline | The app can detect users’ physical presence with periodic scanning of Wi-Fi fingerprints to provide location-based reminders of engaging in limited use with classmates |
| 9 | None | Yes | None | None |
| 10 | None | Yes | None | None |
| 11 | Yes | Yes | Timeline | User can create different use limits for weekdays vs weekends |
| 12 | None | Yes | None | None |
| 13 | None | Yes | Charts | None |
| 14 | None | Yes | Timeline | The app support setting use limit for a group of users |
| 15 | Yes | Yes | Timeline | None |
| 16 | Yes | Yes | Timeline | None |
| 17 | None | Yes | Timeline, charts | None |

Tracking functionality for phone/apps use, format for visualizing the tracked data, and user profiling based on tracked data
