# Supplementary material for: Toward Research-Informed Design Implications for Interventions Limiting Smartphone Use: Functionalities Review of Digital Well-being Apps
Source: JMIR Form Res. 2022 Apr 19;6(4):e31730. doi: 10.2196/31730 (PMC9066336; doi:10.2196/31730)
Supplement: Multimedia Appendix 3 [file formative_v6i4e31730_app3.docx]

| App ID | Setting scope of  limited use | Setting place of limited use - locations | Setting place of limited use WIFI | Setting focus time for offline activi-ties | Visualizing  Time use limits; or time for offline focus activities | Option to  use allowance  beyond  time limit/  focus limit | Option to exclude apps from time limit | Option to disconti-nue tracking when limit reached |
| --- | --- | --- | --- | --- | --- | --- | --- | --- |
| Commercial apps | | | | | | | | |
| 1 | Some apps | None | None | None | Time spent out of time limit; Progress bar gradually filled with color | None | Some Apps | None |
| 2 | None | None | None | Yes | Time unspent out of time limit; Countdown timer | None | None | None |
| 3 | Some apps | None | None | None | Time unspent out of time limit; Progress bar gradually filled with color | Extra time given by parents to children | Some Apps | None |
| 4 | All apps | None | None | None | Time spent out of time limit; Circle gradually filled with color | Phone use allowed after set time limit; with notification: small counter showing the time spent on that app for today | All Apps | None |
| 5 | Some apps | None | None | Yes | Time unspent out of focus time limit; Countdown timer | Two modes for setting focus time limit: strict mode where allowance can be requested when the target app cannot be open until user stops the timer; and normal mode when user can open any app. | Some apps | Yes |
| 6 | Phone | None | None | None | Time unspent out of time limit; Text | The first time when apps are used during the set time limit is free; but the following uses of the apps incur financial penalty | Some Apps | None |
| 7 | All apps | None | None | None | Time spent out of time limit; Text | None | Some Apps | None |
| 8 | Some apps | None | None | None | Time spent out of time limit; Text | None | Some Apps | None |
| 9 | Some apps | Yes | Yes | None | Time spent –tracked only; Text | None | All Apps | None |
| 10 | Some apps | None | None | Yes | Time unspent out of focus time limit; Countdown timer | Only calls allowed for set focus time limit | Some Apps | None |
| 11 | Some apps | None | None | None | Time spent – tracked only; Text | Friction: admin password must be entered in order to use the apps after the set time limit | None | None |
| 12 | Some apps | None | None | None | None | When the set time limit is reached, it can be ignored in 2 ways; to ignore the limit for today (whole day) or to choose “remind me in 15 mins” | Some Apps | None |
| 13 | Phone | None | None | None | Time spent – tracked only; Progress bar gradually filled with color | None | None | None |
| 14 | Phone | None | None | Yes | Time unspent out of time limit; Countdown timer | None | None | None |
| 15 | None | None | None | Yes | Time spent out of time limit; Text | None | None | None |
| 16 | Some apps | None | None | None | None | None | Some Apps | None |
| 17 | All apps | None | None | None | Time spent out of daily usage goal: Text | None | Some Apps | Yes |
| 18 | None | None | None | None | Time spent – tracked only: Text | None | None | None |
| 19 | Some apps | None | None | None | Time spent – tracked only; Text | None | Some Apps | None |
| 20 | Phone | None | None | Yes | None | Only calls allowed for set focus time limit | Some Apps | Yes |
| 21 | Phone | None | None | Yes | None | None | None | None |
| 22 | Some apps | None | None | None | None | None | Some Apps | None |
| 23 | All apps | Yes | None | None | Time spent out of time limit; Progress bar gradually filled with color | None | None | None |
| 24 | None | None | None | None | None | None | None | None |
| 25 | None | None | None | None | None | None | None | None |
| 26 | Phone | None | None | Yes | Time unspent out of focus time: Countdown timer; Circle progressively unfilled with color | Friction: Touch the screen while blocked for 5 sec to access the 30 sec break out of focus time to use the phone | None | Yes |
| 27 | All apps | None | None | None | Time overspent as % of time limit: Text | None | Some Apps | None |
| 28 | None | None | None | None | None | None | None | None |
| 29 | None | None | None | Yes | Time unspent out of time limit; Countdown timer | None | None | Yes |
| 30 | Some apps | None | None | None | None | Option ignore app from set time limit; no further notification for apps’ overuse | Some Apps | Yes |
| 31 | None | None | None | Yes | Time unspent out of time limit; Countdown timer | None | None | None |
| 32 | None | None | None | None | None | None | None | None |
| 33 | None | None | None | Yes | Time unspent out of time limit; Countdown timer | None | None | Yes |
| 34 | None | None | None | Yes | Time unspent out of time limit; Circle progressively unfilled with color | None | None | Yes |
| 35 | Phone, all apps | Yes | None | None | None | Free access for 20 sec; end block with penalty, i.e., ~£4 | Some Apps | None |
| 36 | Some apps | None | None | Yes | Time unspent out of time limit;  Circle progressively unfilled with color | None | Some Apps | Yes |
| 37 | None | None | None | Yes | None | None | None | None |
| 38 | None | None | None | None | None | None | None | None |
| 39 | None | None | None | None | None | None | None | None |
| Academic apps | | | | | | | | |
| 1 | Some apps | None | None | None | None | None | All apps | None |
| 2 | All apps, phone | Yes | None | None | Time spent per app – tracked only; Text, charts | Pop up notification with options: close the app, snooze, or delete | Some apps | Yes |
| 3 | Phone | None | None | None | Time spent out of time limit; Gradually filled with darker color - floating widget: > 50%  (dark yellow), 75% (orange) and 100% (red-maroon) | None | None | Yes |
| 4 | Some apps, phone | None | None | None | None | Users choose either “OK I won’t use it” or “Please, don’t block me again” | Some apps | Yes |
| 5 | None | None | None | None | None | None | None | None |
| 6 | Some apps | None | None | None | Time spent out of time limit; Text, Progress bar gradually filled with color | Users can always request extension for set time limit | Some apps | None |
| 7 | Some apps | None | None | None | Recent usage status: push notification | None | None | None |
| 8 | All apps | Yes | Yes | Yes | Total time unspent out of focus time limit | None | None | Yes |
| 9 | Some apps | None | None | Yes | Time unspent out of time limit; Countdown timer | None | Some apps | Yes |
| 10 | Some apps | None | None | None | None | None | None | None |
| 11 | All apps | None | None | Yes | Time spent out of time limit; Text, Time unspent out of time limit; Countdown timer | When exceeding the limit goal for the first time,  the phone is locked for 1 minute, followed by a 15 minute allowance time. After the 16 mins the lockout duration increases | None | None |
| 12 | None | None |  | None | None | None | None | None |
| 13 | Some apps | None | None | None | None | None | All apps | None |
| 14 | All apps | None | Yes | Yes | Total limit time spent in specific activity e.g. study; Timeline | A cumulative five minutes is allowed after the group start limiting | None | Yes |
| 15 | All apps | None | None | Yes | Time unspent out of time limit; Progress bar gradually unfilled with color | The user can stop limiting if smartphone use is necessary by clicking a give-up button | None | Yes |
| 16 | All apps | None | None | Yes | Time unspent out of time limit; Progress bar gradually filled with color | The user can stop limiting if smartphone use is necessary by clicking a give-up button | None | Yes |
| 17 | All apps | None | None | None | None | None | None | None |

Monitoring functionality: setting use/focus time limits, scope and place of limited use, visualizing time limit, and flexibility through 3 options: use allowance beyond time limit, exclude apps from time limit, and for discontinuing tracking when limit was reached
