# Supplementary material for: Toward Research-Informed Design Implications for Interventions Limiting Smartphone Use: Functionalities Review of Digital Well-being Apps
Source: JMIR Form Res. 2022 Apr 19;6(4):e31730. doi: 10.2196/31730 (PMC9066336; doi:10.2196/31730)
Supplement: Multimedia Appendix 4 [file formative_v6i4e31730_app4.docx]

| App ID | Creating obstacles – types according to force (strong or weak) | Creating obstacles – saliency (explicit or implicit) | Creating obstacles – time (during use, after overuse) | Creating obstacles – social types | Creating obstacles levels tailored to user profile /preference | Creating obstacles – source (app vs user) |
| --- | --- | --- | --- | --- | --- | --- |
| Commercial apps | | | | | | |
| 1 | Strong: strict mode that prevents the child from editing the limits | Explicit; block | After overuse | Parental control | The profile is tailored to specific child by parent | Customized by parent |
| 2 | None | None |  | Social commitment | None | None |
| 3 | Strong: strict mode that prevents the child from editing the limits | Explicit; block | After overuse | Parental control | The profile is tailored by parent to specific child | Customized by parent |
| 4 | Weak: notification on time limit, block app; Strong: phone block. | Explicit; notification, block app, phone block. | After overuse; for not use (blocking phone) | None | 3 levels of challenges: basic, moderate, advanced for specific apps or phone | Automatic |
| 5 | None | None | None | None | None | None |
| 6 | Strong: apps block | Explicit; block | After overuse | None | None | Customized by user |
| 7 | Weak: allows altering the limits | Implicit; screen dimming | After overuse | None | Flexible either take a quiz and tailored to user profile or customized as needed | Customized by user |
| 8 | Weak: users choose the obstacle: push notifications on overuse, pop up warning of overuse or Strong: app block | Explicit; notification & pop up warning, phone block | After overuse | None | Users can limit their usage based on categories of usage (Game, Entertainment, Education, Utility) | Customized by user |
| 9 | Strong: apps block | Explicit; block | During use | None | User choose which app to block | Customized by user |
| 10 | Strong: app block or phone block | Explicit: phone block, apps block | After overuse & for not use (blocking phone) | None | 3 modes: normal, lock mode, strict mode | Customized by user |
| 11 | Strong: apps block or phone block | Explicit: phone block or apps block | During use or after overuse | Parental control | None | Customized by parents |
| 12 | Weak: pop up notification of reaching time limit that can be ignored | Explicit: pop up notification | After overuse | None | User preferences | Automatic |
| 13 | None | None | None | None | None | None |
| 14 | Strong: phone block | Explicit: phone block | For not use | None | User preferences | Customized by user |
| 15 | None | None | None | None | None | None |
| 16 | Strong: apps block | Explicit: apps block | After overuse | None | User preference: daily limit, scheduled limit, or timer | Automatic |
| 17 | Weak: push notification reminding users of today’s usage timer | Explicit: push notification | After overuse | None | None | Automatic |
| 18 | None | None |  | None | None | None |
| 19 | Strong: apps block | Explicit; block | During use | None | None | Automatic |
| 20 | Strong: phone block | Explicit: phone block | For not use | None | User preference or tailored to user profile: easy, medium, hard, grand master | Customized by user |
| 21 | Strong: phone block | Explicit; block | During use | None | None | Automatic |
| 22 | Strong: apps block | Explicit: apps block | After overuse | None | User preference | Customized by user |
| 23 | None | None | None | None | None | None |
| 24 | None | None | None | None | None | None |
| 25 | None | None | None | None | None | None |
| 26 | Strong: phone block | Explicit: phone block | For not use | None | None | Customized by user |
| 27 | None | None | None | None | None | None |
| 28 | None | None | None | None | None | None |
| 29 | None | None | None | None | None | None |
| 30 | Strong: apps block | Explicit: apps block | After overuse | Parental control | None | Customized by parents |
| 31 | None | None | None | None | None | None |
| 32 | None | None | None | None | None | None |
| 33 | None | None | None | None | None | None |
| 34 | None | None | None | None | None | None |
| 35 | Strong: apps block | Explicit: apps block | Scheduled or after overuse | None | None | Customized by user |
| 36 | Strong: app block | Explicit: apps block | After overuse | None | Instant block or users can profile blocking | Customized by user |
| 37 | None | None | None | None |  | None |
| 38 | None | None | None | None | None | None |
| 39 | None | None | None | None | None | None |
| Academic apps | | | | | | |
| 1 | Strong: app block | Explicit: app block | During use | None | User preference | Customized by user |
| 2 | Weak: pop un notification Strong: app block, phone block | Explicit: notification, app or phone block | During & after use | None | User preference | Customized by user |
| 3 | Weak: small floating widget turn to red-maroon color, | Explicit: red-maroon floating widget | After overuse | None | User preference | Automatic |
| 4 | Weak: pop up notification | Explicit: notification | After overuse | None | User preference | Automatic |
| 5 | None | None | None | None | None | None |
| 6 | Weak: pop up notification | Explicit: notification | After overuse | None | User preference | Customized by user |
| 7 | Weak: gentle vibrations every five seconds | Implicit: vibration | After overuse | None | User preference | Automatic |
| 8 | Strong: phone block Weak: notifications muted | Explicit: phone block, notifications muted | During use | classmates | User preference | Automatic |
| 9 | Weak: app block, deactivated if user stops the timer, mute notifications | Explicit: app block, mute notifications | During use | None | User preference | Customized by user |
| 10 | Prior interaction. Weak: entering 5 random numbers displayed Strong: entering more random number displayed | Explicit: friction; entering random numbers | Before use | None | User preference | Automatic |
| 11 | Weak: phone block followed by allowance time  Strong: phone block until midnight | Explicit: phone block; friction: password must be entered in order to use the apps after the set time limit | After overuse | None | User preference | Customized by user |
| 12 | Weak: press ok to launch app Strong: enter 30 random digits displayed to launch app | Explicit: press ok, enter 30 random digits prior to launching specific app | Before use | None | User preference | Automatic |
| 13 | Strong: app block | Explicit: app block | During use & after overuse | None | The app provides rules for the user to choose from: specific daytimes, number of launches, usage time, activity based, some time, forever | Customized by user |
| 14 | Weak: mute all notification | Explicit: mute notifications | During use | None | None | Automatic |
| 15 | Strong: app block | Explicit: app block | During use | None | limiting mode overrides  all apps except for checking a notification drawer | Automatic |
| 16 | Strong: app block | Explicit: ap block | During use | None | None | Automatic |
| 17 | Strong: app block | Explicit: app block | After overuse | None | The app is tailored to elementary to high-school students | Automatic |

Interventions for limiting use: creating obstacles for limiting use differing in force, saliency, temporality, sociality, user profile, and source
