# Supplementary material for: Toward Research-Informed Design Implications for Interventions Limiting Smartphone Use: Functionalities Review of Digital Well-being Apps
Source: JMIR Form Res. 2022 Apr 19;6(4):e31730. doi: 10.2196/31730 (PMC9066336; doi:10.2196/31730)
Supplement: Multimedia Appendix 5 [file formative_v6i4e31730_app5.docx]

| App ID | Notifications for reaching use limits | Notifications for reaching use limit - type | Notifications for reaching phone time limit on digital wellbeing app | Notifications on reaching app time limit - on those specific apps | Screen dimming for reaching use limit | Daily reminders to review tracked data |
| --- | --- | --- | --- | --- | --- | --- |
| Commercial apps | | | | | | |
| 1 | None | None | None | None | None | None |
| 2 | None | None | None | None | None | None |
| 3 | None | None | None | None | None | None |
| 4 | Yes | Explicit: push notification | Yes: time up | None | None | None |
| 5 | None | None | None | None | None | None |
| 6 | Yes | Explicit: pop up notification | Yes: lock icon next to app name | Yes: pop up notification and closing the app | None | None |
| 7 | Yes | Implicit: screen dimming | Yes: time up | None | Yes | None |
| 8 | Yes | Explicit: push or pop up notification | None | Yes: push notification reminder | None | Yes |
| 9 | None | None | None | None | None | None |
| 10 | Yes | Explicit: pop up notification | None | Yes: pop up notification covers the app | None | None |
| 11 | None | None | None | None | None | None |
| 12 | Yes | Explicit: pop up notification | None | Yes: transparent pop up notification | None | None |
| 13 | Yes | Explicit: progress bar filled with color | Yes: progress bar filled with color | None | None | None |
| 14 | None | None | None | None | None | None |
| 15 | None | None | None | None | None | None |
| 16 | Yes | Explicit: pop up notification | None | Yes: pop up notification covers the app | None | None |
| 17 | Yes | Explicit: push notification | None | None | None | Yes |
| 18 | None | None | None | None | None | None |
| 19 | None | None | None | None | None | Yes |
| 20 | None | None | None | None | None | None |
| 21 | None | None | None | None | None | None |
| 22 | Yes | Explicit: push notification | None | Yes: pop up notification and closing the app | None | None |
| 23 | None | None | None | None | None | Yes |
| 24 | None | None | None | None | None | None |
| 25 | None | None | None | None | None | None |
| 26 | None | None | None | None | None | None |
| 27 | None | None | None | None | None | Yes |
| 28 | None | None | None | None | None | None |
| 29 | None | None | None | None | None | None |
| 30 | Yes | Explicit | Yes | Yes | None | Yes |
| 31 | None | None | None | None | None | None |
| 32 | None | None | None | None | None | None |
| 33 | None | None | None | None | None | None |
| 34 | None | None | None | None | None | None |
| 35 | Yes | Explicit: pop up notification | None | Yes: pop up notification and closing the app | None | None |
| 36 | Yes | Explicit: pop up notification | None | Yes: pop up notification and closing the app | None | None |
| 37 | None | None | None | None | None | None |
| 38 | None | None | None | None | None | None |
| 39 | None | None | None | None | None | None |
| Academic apps | | | | | | |
| 1 | None | None | None | None | None | None |
| 2 | Yes | Explicit: pop up notification | Yes | Yes | None | None |
| 3 | Yes | Explicit: notification from the conversational agent (chatbot) | Yes | None | None | Yes |
| 4 | Yes | Explicit: pop up notification | None | Yes | None | None |
| 5 | None | None | None | None | None | None |
| 6 | Yes | Explicit: pop up notification | None | Yes | None | None |
| 7 | Yes | Explicit: gentle vibration | None | Yes | None | None |
| 8 | None | None | None | None | None | None |
| 9 | None | None | None | None | None | None |
| 10 | None | None | None | None | None | None |
| 11 | Yes | Explicit: notification dialog | None | None | None | None |
| 12 | None | None | None | None | None | None |
| 13 | Yes | Explicit: pop up notification | None | Yes | None | None |
| 14 | None | None | None | None | None | None |
| 15 | None | None | None | None | None | None |
| 16 | None | None | None | None | None | None |
| 17 | Yes | Explicit: pop up notification | None | None | None | None |

Interventions for limiting use: supporting awareness for reaching the set limit of use through different notification types, screen diming, and daily reminders.
