# Supplementary material for: Toward Research-Informed Design Implications for Interventions Limiting Smartphone Use: Functionalities Review of Digital Well-being Apps
Source: JMIR Form Res. 2022 Apr 19;6(4):e31730. doi: 10.2196/31730 (PMC9066336; doi:10.2196/31730)
Supplement: Multimedia Appendix 6 [file formative_v6i4e31730_app6.docx]

| App ID | Supporting focused attention - training | Supporting focused attention – white noise |
| --- | --- | --- |
| Commercial apps | | |
| 1 | None | None |
| 2 | Yes | Yes |
| 3 | None | None |
| 4 | None | None |
| 5 | Yes | Yes |
| 6 | None | None |
| 7 | None | None |
| 8 | None | None |
| 9 | None | None |
| 10 | None | None |
| 11 | None | None |
| 12 | None | None |
| 13 | None | None |
| 14 | None | None |
| 15 | Yes | None |
| 16 | None | None |
| 17 | None | None |
| 18 | None | None |
| 19 | None | None |
| 20 | None | None |
| 21 | None | None |
| 22 | None | None |
| 23 | None | None |
| 24 | None | None |
| 25 | None | None |
| 26 | Yes | None |
| 27 | None | None |
| 28 | None | None |
| 29 | Yes | Yes |
| 30 | None | None |
| 31 | Yes | None |
| 32 | None | None |
| 33 | Yes | Yes |
| 34 | None | None |
| 35 | None | None |
| 36 | None | None |
| 37 | Yes | Yes |
| 38 | None | None |
| 39 | None | None |
| Academic apps | | |
| 1 | None | None |
| 2 | None | None |
| 3 | None | None |
| 4 | None | None |
| 5 | None | None |
| 6 | None | None |
| 7 | None | None |
| 8 | Yes | None |
| 9 | Yes | None |
| 10 | None | None |
| 11 | Yes | None |
| 12 | None | None |
| 13 | None | None |
| 14 | None | None |
| 15 | Yes | None |
| 16 | Yes | None |
| 17 | None | None |

Interventions for limiting use: supporting focused attention through training or white noise
