# Supplementary material for: Toward Research-Informed Design Implications for Interventions Limiting Smartphone Use: Functionalities Review of Digital Well-being Apps
Source: JMIR Form Res. 2022 Apr 19;6(4):e31730. doi: 10.2196/31730 (PMC9066336; doi:10.2196/31730)
Supplement: Multimedia Appendix 7 [file formative_v6i4e31730_app7.docx]

| App ID | Motivation for keeping within use limit - types | Motivation for keeping within use limit - rewarding  content | Motivation for keeping within use limit - punitive  content | Motivation for keeping within limits: educational content, quotes | Providing access to social support - type | Social support: coope-ration | Social support: compe-tition | Social support: recog-nition |
| --- | --- | --- | --- | --- | --- | --- | --- | --- |
| Commercial apps | | | | | | | | |
| 1 | None | None | None | None | Family | None | None | None |
| 2 | Punitive, Rewarding | Virtual coins that can be used later to unlock paid features, Plant virtual and real trees on Earth | If 25 min set time for offline activity is not met, 1 tree in forest withers | None | Friends, wider social network | Yes | Yes | Yes |
| 3 | Rewarding | Parent reward the child extra time for good behavior | None | None | Family | Yes | None | None |
| 4 | Rewarding | Badges (Bronze, Silver, Gold) | None | Provide motivational stories written by others | None | None | None | None |
| 5 | Rewarding | Virtual sunlight is generated that can be collected as points after 24 hours | None | None | Wider social network | Yes: join user groups | Yes: ranks based on focus time in group | Yes: ranks based on focus time - among other users of the app |
| 6 | None | None | None | None | None | None | None | None |
| 7 | Punitive | None | Screen dimming | 8 days course for phone/life balance | Friends | None | Yes | Yes |
| 8 | Rewarding | Inspiring quotations | None | Option for user generated motivational text that appears when exceeding limit | None | None | None | None |
| 9 | None | None | None | None | None | None | None | None |
| 10 | None | None | None | Option for user generated motivational text that appears when the app is blocked | None | None | None | None |
| 11 | Punitive | None | Parents can lock the phone of the child with a button press | None | Family | None | None | None |
| 12 | None | None | None | None | None | None | None | None |
| 13 | None | None | None | None | None | None | None | None |
| 14 | None | None | None | None | None | None | None | None |
| 15 | None | None | None | None | None | None | None | None |
| 16 | None | None | None | None | None | None | None | None |
| 17 | None | None | None | Option for user generated motivational text that appears when exceeding limit | None | None | None | None |
| 18 | None | None | None | None | None | None | None | None |
| 19 | Rewarding | Levels: bronze, silver, gold, platinum, iron, titanium, vibranium, adamantium | None | None | None | None | None | None |
| 20 | Rewarding | Points that can be used at Google Play Games | None | Suggestions for offline activities, i.e., "Ride a bike", "Look at the clouds", "Daydream", "Write a letter", shown on the screen under the time left to use the phone | None | None | None | None |
| 21 | None | None | None | None | None | None | None | None |
| 22 | None | None | None | None | None | None | None | None |
| 23 | None | None | None | None | None | None | None | None |
| 24 | None | None | None | Launcher showing the minimum number of apps according to the user needs | None | None | None | None |
| 25 | None | None | None | None | None | None | None | None |
| 26 | None | None | None | None | None | None | None | None |
| 27 | None | None | None | None | None | None | None | None |
| 28 | None | None | None | None | None | None | None | None |
| 29 | None | None | None | None | None | None | None | None |
| 30 | None | None | None | None | Family | None | None | None |
| 31 | None | None | None | None | None | None | None | None |
| 32 | Rewarding, punitive | Virtual town will be built | If using phone during bed time the user’s virtual building will collapse | None | Friends | Yes | Yes | Yes |
| 33 | None | None | None | When user feels distracted, can click in the circle and the apps counts the number of distractions and save it in history. It does not support specifying the type of distraction | None | None | None | None |
| 34 | None | None | None | None | None | None | None | None |
| 35 | Punitive | None | Paying a penalty to end lockouts early | None | None | None | None | None |
| 36 | None | None | None | Option for user generated motivational text that appears when the app is blocked | None | None | None | None |
| 37 | Rewarding | Points | None | None | Family, friends, wider social network | Yes: Engage together in focus time | Yes: add friends and compete to win | Yes: unlock rewards |
| 38 | None | None | None | None | None | None | None | None |
| 39 | None | None | None | None | None | None | None | None |
|  | **Academic apps** | | | | | | | |
| 1 | None | None | None | None | None | None | None | None |
| 2 | None | None | None | None | None | None | None | None |
| 3 | None | None | None | None | None | None | None | None |
| 4 | None | None | None | None | None | None | None | None |
| 5 | None | None | None | None | Family | Yes: parent intervention when the child uses the video platform | None | None |
| 6 | None | None | None | None | None | None | None | None |
| 7 | None | None | None | None | None | None | None | None |
| 8 | None | None | None | None | classmates | None | Yes: ranks based on focus time | Yes: ranks based on focus time among classmates |
| 9 | None | None | None | None | None | None | None | None |
| 10 | None | None | None | None | None | None | None | None |
| 11 | None | None | None | None | None | None | None | None |
| 12 | None | None | None | None | None | None | None | None |
| 13 | None | None | None | None | None | None | None | None |
| 14 | Rewarding | Points | None | None | Friends | Yes: limit together and focus on specific task | None | None |
| 15 | Rewarding | Points | None | None | Family | None | Yes: ranks based on points | Yes: summary of family limit state |
| 16 | Rewarding | Points | None | None | Friends, Family | None | Yes: ranks based on points | Yes: summary of group limit state |
| 17 | None | None | None | None | None | None | None | None |

Interventions for limiting use: Supporting motivation to keep within limited use involving different types and content, as well as social support of different types involving cooperation, competition and associated social recognition
